# Identification of potential targets of cinnamon for treatment against Alzheimer’s disease-related GABAergic synaptic dysfunction using network pharmacology

**DOI:** 10.1038/s41598-022-24378-0

**Published:** 2022-11-19

**Authors:** Dongdong Qian, Qixue Wang, Siyuan Lin, Ying Li, Xinyi Gu, Chenyi Xia, Ying Xu, Ting Zhang, Li Yang, Qianfu Wu, Jijia Sun, Yi Liu, Mingmei Zhou

**Affiliations:** 1 Department of Geriatrics, Shanghai Municipal Hospital of Traditional Chinese Medicine, Shanghai University of Traditional Chinese Medicine, Shanghai, 200071 China; 2 Institute of Interdisciplinary Integrative Medicine Research, Shanghai University of Traditional Chinese Medicine, 1200 Cailun Road, Pudong District, Shanghai, 201203 China; 3 School of Public Health, Shanghai University of Traditional Chinese Medicine, Shanghai, 201203 China; 4 Department of Physiology, School of Basic Medical Sciences, Shanghai University of Traditional Chinese Medicine, Shanghai, 201203 China; 5 Department of Mathematics and Physics, School of Pharmacy, Shanghai University of Traditional Chinese Medicine, Shanghai, 201203 China

**Keywords:** Blood-brain barrier, Cognitive ageing

## Abstract

Cinnamon aqueous extract’s active substance base remains unclear and its mechanisms, mainly the therapeutic target of anti-Alzheimer’s disease (AD)-related GABAergic synaptic dysfunction, remain unclear. Here, 30 chemical components were identified in the aqueous extract of cinnamon using LC/MS; secondly, we explored the brain-targeting components of the aqueous extract of cinnamon, and 17 components had a good absorption due to the blood–brain barrier (BBB) limitation; thirdly, further clustering analysis of active ingredient targets by network pharmacology showed that the GABA pathway with GABRG2 as the core target was significantly enriched; then, we used prominent protein–protein interactions (PPI), relying on a protein-metabolite network, and identified the GABRA1, GABRB2 and GABRA5 as the closest targets to GABRG2; finally, the affinity between the target and its cognate active compound was predicted by molecular docking. In general, we screened five components, methyl cinnamate, propyl cinnamate, ( +)-procyanidin B2, procyanidin B1, and myristicin as the brain synapse-targeting active substances of cinnamon using a systematic strategy, and identified GABRA1, GABRB2, GABRA5 and GABRG2 as core therapeutic targets of cinnamon against Alzheimer's disease-related GABAergic synaptic dysfunction. Exploring the mechanism of cinnamon’ activities through multi-components and multiple targets strategies promise to reduce the threat of single- target and symptom-based drug discovery failure.

The main pathological characteristics of Alzheimer’s disease (AD) are the accumulation of β-amyloid peptide (Aβ) and neurofibrillary tangles (NFTs) composed of hyperphosphorylated tau. Meanwhile, the synaptic deficit is comprehensively recognized as a pathogenic event in AD. Glutamatergic and GABAergic are the two main synaptic types in the central nervous system, which provide excitatory and inhibitory outputs, respectively, and a large body of data suggests that glutamatergic systems are often damaged when disease occurs^1^. Nevertheless, many lines of evidence unveil whether the disruption of the default neuronal network leads to memory deficit, which engenders distinct changes to GABAergic circuits, either primarily or as a compensatory response to excitotoxicity, with a concomitant loss of AD by disrupting overall network function^2^. The GABAergic system consists of GABA, GABA transporters, GABAergic receptors, and GABAergic neurons. Changes in any of these components may lead to dysfunction of the central nervous system^3^.

Cinnamon has a long history of medicinal use, and available in vitro and in vivo evidence suggests anti-inflammatory, antibacterial, antioxidant, antitumor, cardiovascular, cholesterol-lowering, and immunomodulatory effects^4^. Ultra-performance liquid chromatography-quadrupole-time-of-flight tandem mass spectrometry (UPLC-Q-TOF–MS/MS) is a combined technique that perfectly integrates the high-efficiency online separation of chromatography with the high sensitivity and selectivity of mass spectrometry^5^. It can accurately and quickly conduct a qualitative analysis of the chemical components of natural herbs^6^. The aqueous extract of Ceylon cinnamon was found to inhibit tau aggregation and filament formation, which are characteristics of AD^7^. Therefore, it is particularly important to identify the small molecules in cinnamon aqueous extracts that are active against diseases. Cinnamaldehyde is the main active component of cinnamon aqueous extracts, which may be further converted to cinnamyl alcohol, methyl cinnamate, and cinnamic acid in vivo^8^. Methyl cinnamate was reported to be one of the active components of the standardized ethanolic extract of Mikania glomerata (EPMG), and the findings suggest that the anxiolytic effect displayed by the ethanolic extract of EPMG may be mediated by the GABAergic system and can upregulate GABA concentrations and decrease glutamate and aspartate levels in the hippocampus of mice, which could directly and/or indirectly exhibit its anxiolytic effects^9^.

The prevalence of anxiety symptoms in AD is about 40%, and it may be a precursor to AD. Anxiety often occurs early in AD, especially in patients with mild cognitive impairment (MCI), mild dementia or early-onset disease, and facilitates the development and transition from MCI to dementia^10^. Cinnamaldehyde has been reported in the literature to reduce anxiety-related behaviors in mice^11^. Cinnamon aqueous extracts were identified to contain cinnamaldehyde, trans-cinnamaldehyde, etc. Therefore, this paper attempts to identify the core components and key targets of cinnamon aqueous extracts with anti-AD-related GABAergic synapse deficits using a network pharmacology method to elucidate the anti-anxiety mechanism. Then the flow chart of this paper is shown in Fig. 1.

**Figure 1:**
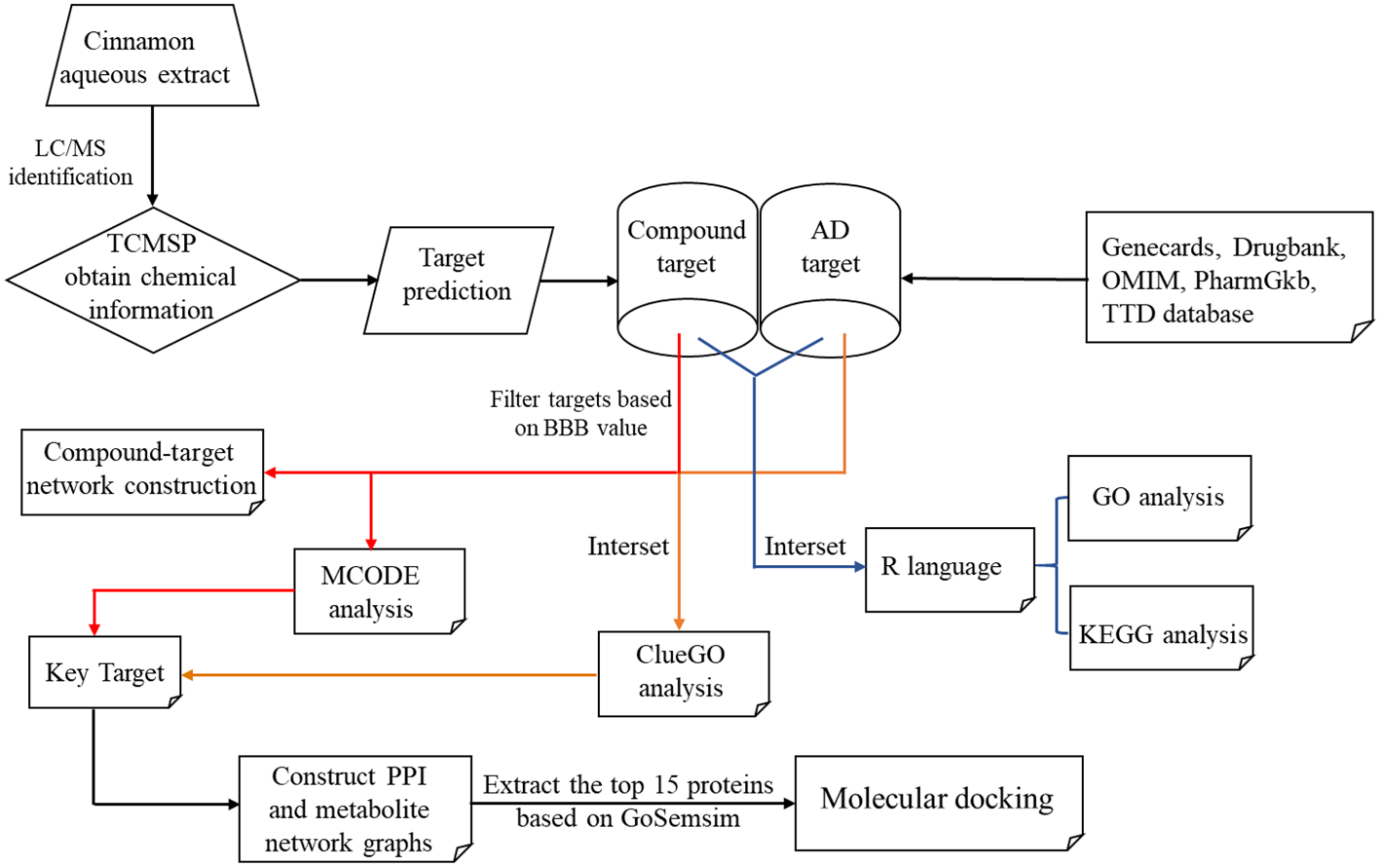
Research strategy to find AD-related GABAergic synapse dysfunction by identifying the active ingredients of cinnamon aqueous extracts.

## Results

### Preparation of cinnamon aqueous extracts and LC/MS analysis

Weigh 10.0191 g of the herb, add 100 mL of distilled water, reflux the extract for 2 h, then extract the extract while hot, and place the aqueous extract of cinnamon in an oven at 80 °C until it is steamed into an infusion, and weigh it to obtain 0.7012 g. 207.7 mg of dry extract was then weighed and dissolved in 2 mL of 50% methanol solution to obtain a sample solution with a concentration of 103.85 mg/mL, which was filtered through a 0.22 μm nylon. The sample solution was filtered through a 0.22 μm nylon membrane and 1 mL of supernatant were prepared for sampling.

### Identification and characterization of cinnamon aqueous extracts components

**Figure 2:**
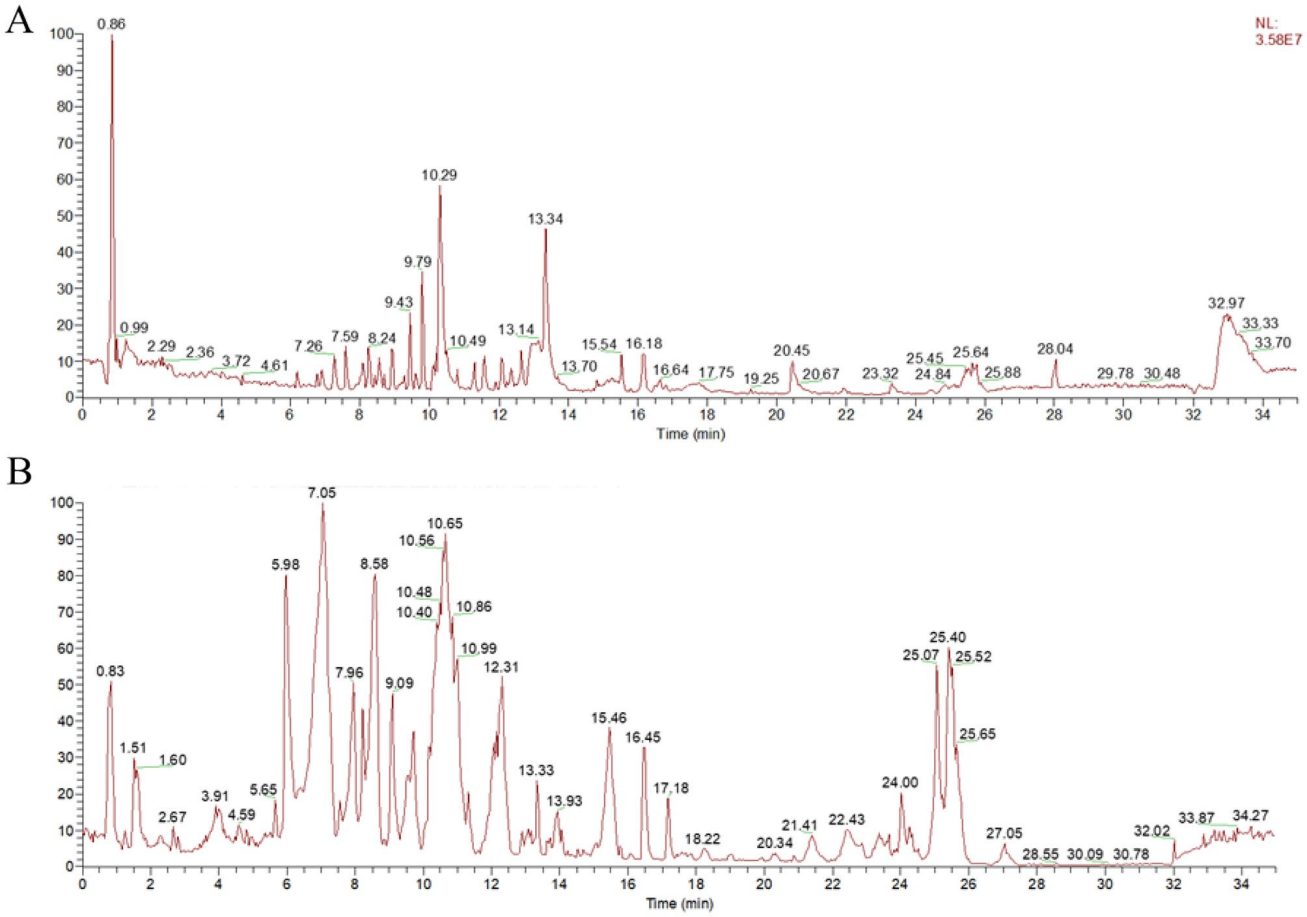
Total ion flow diagram of cinnamon aqueous extract in positive (**A**) and negative (**B**) ion modes.

Mass spectra were obtained in both positive and negative ion modes, and representative total ion chromatograms are shown in Fig. 2. Import the data into the analysis software, and perform qualitative analysis on the ions whose mass error does not exceed 5 ppm, the isotopic distribution is correct, and the ions containing secondary fragments are the target ions. Quickly screen compounds by targeted and non-targeted peak-finding methods, and then use mzVault, mzCloud, ChemSpider and other databases for comparison, or refer to mass spectrometry fragment ion analysis and inference in the literature for preliminary identification. A total of 44 components were screened by targeted and non-targeted peak search methods, and then 30 components were obtained by intersection with TCMSP. As shown in Table 1, 30 chemical components in cinnamon were initially identified, including 13 acids, 5 aldehydes, 3 esters, 2 phenols and 6 other classes. Next, an accurate structure identification was needed in combination with references and other structure identification methods. Among them, cinnamaldehyde, cinnamic acid, cinnamyl alcohol, 4-methoxycinnamaldehyde, palmitoleic acid, methyl cinnamate, coumarin, procyanidin B1, procyanidin C1 were the main components.

**Table 1:** Identification of the chemical composition of cinnamon aqueous extracts by LC–MS.

Mol ID	Compound name	Formula	OB (%)	BBB	[M + H]^+^/[M–H]^−^(m/z)	Mass error (ppm)	Fragment mass (m/z)	Retention time (min)
MOL001739	Palmitoleic acid	C_16_H_30_O_2_	35.78	0.88	253.2164 [M‒H]^−^	− 4.8	169.03305, 96.96008, 82.70850	23.848
MOL000305	Lauric acid	C_12_H_24_O_2_	23.59	1.1	199.1695 [M‒H]^−^	− 4.7	147.39396, 94.53233	20.39
MOL000105	Protocatechuic acid	C_7_H_6_O_4_	25.37	− 0.17	153.0189 [M‒H]^−^	− 2.5	138.03209, 109.02942, 89.02420	1.694
MOL000114	Vanillic acid	C_8_H_8_O_4_	35.47	0.09	167.0344 [M‒H]^−^	− 2.4	152.01117, 108.02143, 91.01884	3.713
MOL002049	Ferulaldehyde	C_10_H_10_O_3_	49.26	0.58	177.0553 [M‒H]^−^	− 3.8	162.03226, 133.06598	10.403
MOL002295	Cinnamic acid	C_9_H_8_O_2_	19.68	0.96	193.0500 [M‒H]^−^	− 3.5	149.06070, 134.03729, 117.03455	7.558
MOL002225	Cinnamyl alcohol	C_9_H_10_O	38.35	1.09	133.0657 [M‒H]^−^	− 2.2	115.92085, 99.92255, 74.51443	11.893
MOL001456	Citric acid	C_6_H_8_O_7_	56.22	− 1.38	191.0552 [M‒H]^−^	− 1.8	129.01930, 111.00871, 87.00870, 85.02945	1.091
MOL000513	Gallic acid	C_7_H_6_O_5_	31.69	− 0.54	169.0136 [M‒H]^−^	− 3	125.06057, 97.03084, 81.03530	1.238
MOL000771	p-Hydroxy-cinnamic acid	C_9_H_8_O_3_	43.29	0.13	163.0398 [M‒H]^−^	− 3.1	145.06601, 119.05019	6.583
MOL000103	4-Hydroxybenzoic acid	C_7_H_6_O_3_	30.15	0.21	137.0241 [M‒H]^−^	− 2.8	108.02285, 79.41434, 70.05586, 61.27248	5.849
MOL000219	Benzoic acid	C_7_H_6_O_2_	31.55	0.84	121.0293 [M‒H]^−^	− 1.1	109.02670, 92.02802, 52.66825	7.27
MOL000360	Ferulic acid	C_10_H_10_O_4_	39.56	− 0.03	193.0500 [M‒H]^−^	− 4.5	178.02681, 149.06070, 137.02444, 117.03455	7.558
MOL001837	Methyl 4-hydroxycinnamate	C_10_H_10_O_3_	20.14	0.5	177.0551 [M‒H]^−^	− 3.8	145.02945, 119.05059, 93.03439	9.922
MOL004102	2-Hydroxycinnamic acid	C_9_H_8_O_3_	53.6	0.28	163.0396 [M‒H]^−^	− 3.2	142.75320, 119.05009, 104.70478, 72.61282	8.61
MOL001807	Syringic acid	C_9_H_10_O_5_	47.78	0.1	197.0449 [M‒H]^−^	− 4.5	153.01913, 137.02437, 125.02437	2.338
MOL000431	Coumarin	C_9_H_6_O_2_	29.17	1.3	147.0443 [M + H]^+^	− 1.7	119.04967, 103.05430, 91.05426	10.302
MOL000250	trans-Cinnamaldehyde	C_9_H_8_O	27.21	1.45	133.0649 [M + H]^+^	− 1.1	105.06988, 77.03823, 55.01781	13.357
MOL000704	Styrene	C_8_H_8_	29.55	2	105.0698 [M + H]^+^	− 1.8	95.04926, 77.03852, 59.47728	13.314
MOL000261	Myristicin	C_11_H_12_O_3_	17.99	1.14	175.0757 [M + H]^+^	− 0.2	133.06520, 117.007018, 107.04935, 91.05431	10.386
MOL002003	( −)-Caryophyllene oxide	C_15_H_24_O	32.67	1.76	222.0467 [M + H]^+^	0.8	203.17975, 177.12729, 147.11705, 133.10139	14.505
MOL000991	Cinnamaldehyde	C_9_H_8_O	31.99	1.48	133.06479 [M + H]^+^	0	133.06479	4.89
MOL003530	2-Hydroxycinnamaldehyde	C_9_H_8_O_2_	26.52	1.18	149.0496 [M + H]^+^	− 0.1	121.06494, 104.05760, 77.03910, 55.01800	10.264
MOL007283	( +)-Procyanidin B2	C_30_H_26_O_12_	3.01	− 2.02	579.1493 [M + H]^+^	− 0.6	299.05600, 163.03934, 127.03913	6.897
MOL000874	Paeonol	C_9_H_10_O_3_	28.79	0.84	167.0706 [M + H]^+^	0.7	155.07054, 137.05992, 109.06483	15.47
MOL000004	Procyanidin B1	C_30_H_26_O_12_	67.87	− 1.97	579.1493 [M + H]^+^	− 0.6	299.05600, 287.05569, 163.03934, 127.03913	6.89
MOL013205	4-Methoxycinnamaldehyde	C_10_H_10_O_2_	59.64	0.98	163.0753 [M + H]^+^	− 1.4	135.08061, 121.06494, 107.04929	6.597
MOL000396	( +)-syringaresinol	C_22_H_26_O_8_	3.29	− 0.34	418.0781 [M + H]^+^	0.2	375.80878, 264.23254, 246.22183, 167.07042	10.578
MOL000249	Methyl cinnamate	C_10_H_10_O_2_	18.42	1.3	163.0755 [M + H]^+^	− 1.4	135.08061, 121.06494, 107.04929, 77.03874	6.578
MOL003774	Propyl cinnamate	C_12_H_14_O_2_	39.22	1.22	191.0854 [M + H]^+^	0.7	149.09644, 131.08583, 79.05413	8.197

### Cinnamon aqueous extracts active ingredient screening and target collection of cinnamon and AD

**Figure 3:**
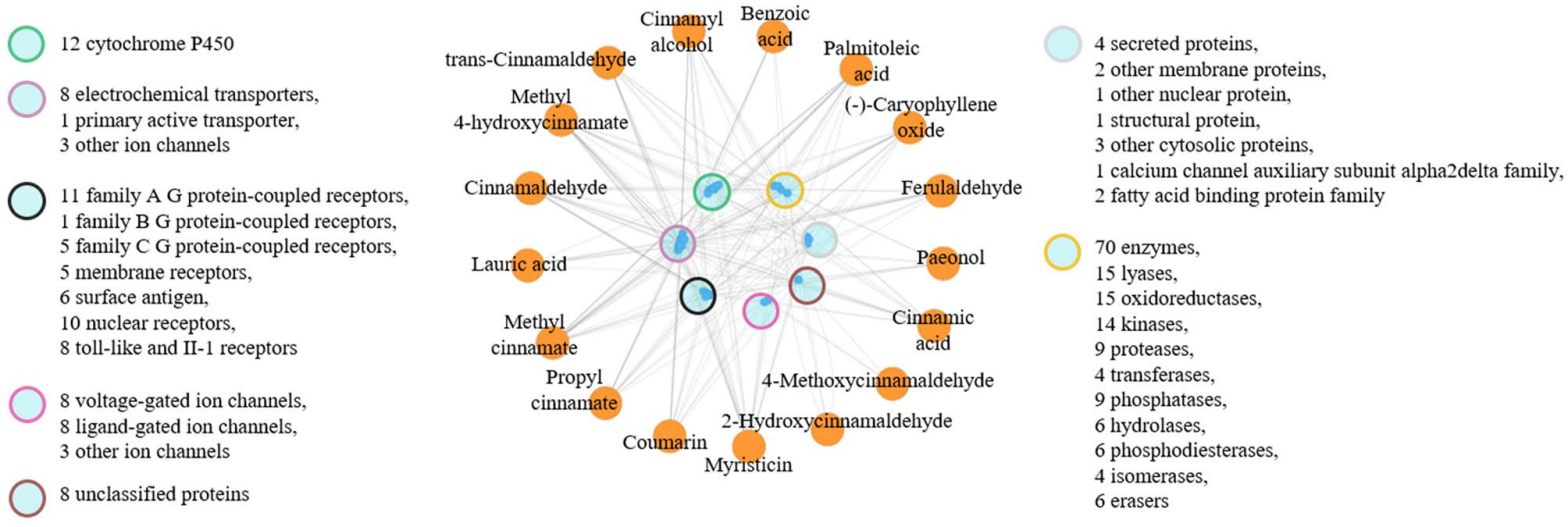
Analysis of cerebral cinnamon targets and corresponding components. The orange circles in the figure represent the active ingredients in the cinnamon water extract that can penetrate the BBB, and the circles with different colors in the middle represent different target types. The entire network graph represents the interaction between the compound and the target.

After screening the swiss ADME website, 26 compounds have a high gastrointestinal absorption rate. According to the literature search, procyanidin B1 and procyanidin B2 were also found to have strong pharmacological effects after metabolism in vivo, so these two chemical components were re-added to the chemical composition information database, with a total of 28 compounds. Among them, there were 17 compounds with BBB values greater than 0.3. From the swiss target prediction and after screening and removing duplicate values, 432 total cinnamon targets and 381 cinnamon targets with BBB values greater than 0.3 were obtained. The types of these 381 targets were further analyzed (Fig. 3). 1626 human AD genes were obtained from five databases, namely Genecards, Drugbank, OMIM, PharmGkb, and TTD. The number of intersections between total cinnamon targets and AD genes was 191. The number of intersections between cinnamon brain targets and AD genes was 169, respectively, obtained by mapping relationship analysis.

### Enrichment analysis of cinnamon aqueous extracts and AD intersection targets

**Figure 4:**
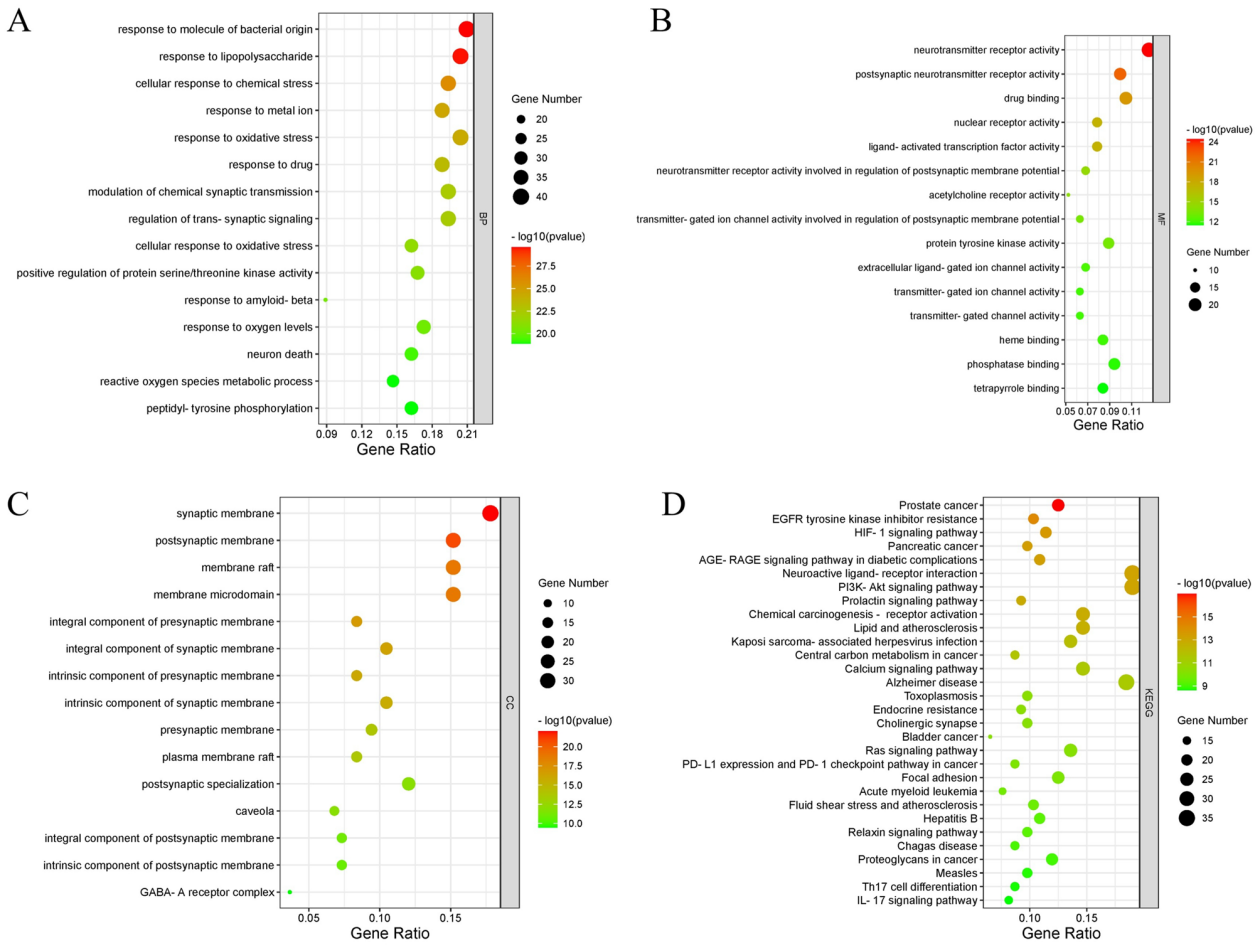
GO and KEGG pathway enrichment analysis of cinnamon and AD intersection targets. (**A**-**C**) GO-BP, MF, CC analyses respectively. (**D**) KEGG analysis.

Running the clusterProfiler, a package in R, the above 191 candidate targets were assessed by enrichment analysis. The outcomes of GO enrichment analysis showed that 191 targets were prominently collected in 2976 GO terms (*p* value < 0.05), including 2625 in BP, 115 in CC, and 236 in MF. According to the GO-BP result, these targets were related to the response to molecules of bacterial origin (confirmed by Ruwizhi et al.^12^), response to lipopolysaccharides, cellular response to chemical stress, response to metal ions, response to oxidative stress (confirmed by Yoo et al.^13^), response to drugs, regulation of chemical synaptic transmission, regulation of trans-synaptic signaling, cellular response to oxidative stress, response to Aβ response, response to oxygen levels, neuronal death, and reactive oxygen metabolic processes (Fig. 4A). GO-CC analyses were mainly enriched in the synaptic membrane (confirmed by Yokoyama et al.^14^), postsynaptic membrane, and GABA-A receptor complex (confirmed by Wei et al.^15^), and organic components of the presynaptic membrane (Fig. 4B). In addition, in terms of GO-MF, these targets involve neurotransmitter receptor activity (confirmed by Wei et al.^15^), postsynaptic neurotransmitter receptor activity, drug binding, nuclear receptor activity (confirmed by Tuzcu et al.^16^), ligand-activated transcription factor activity, activated transcription factor activity (confirmed by Li et al.^17^), and acetylcholine receptor activity (confirmed by Boudry et al.^18^) (Fig. 4C). In addition, an overall of 161 relevant pathways was identified according to Kyoto Encyclopedia of Genes and Genomes (KEGG) analysis (*p* < 0.05), and the first 30 KEGG pathways are also shown (Fig. 4D), mainly including prostate cancer, resistance to EGFR tyrosine kinase inhibitors, HIF-1 signaling pathway, neuroactive ligand-receptor interaction, lipids and atherosclerosis, calcium signaling pathway, Alzheimer’s disease and other pathways.

### Clustering analysis of cinnamon aqueous extracts brain compound targets and enrichment analysis of AD target intersection

**Figure 5:**
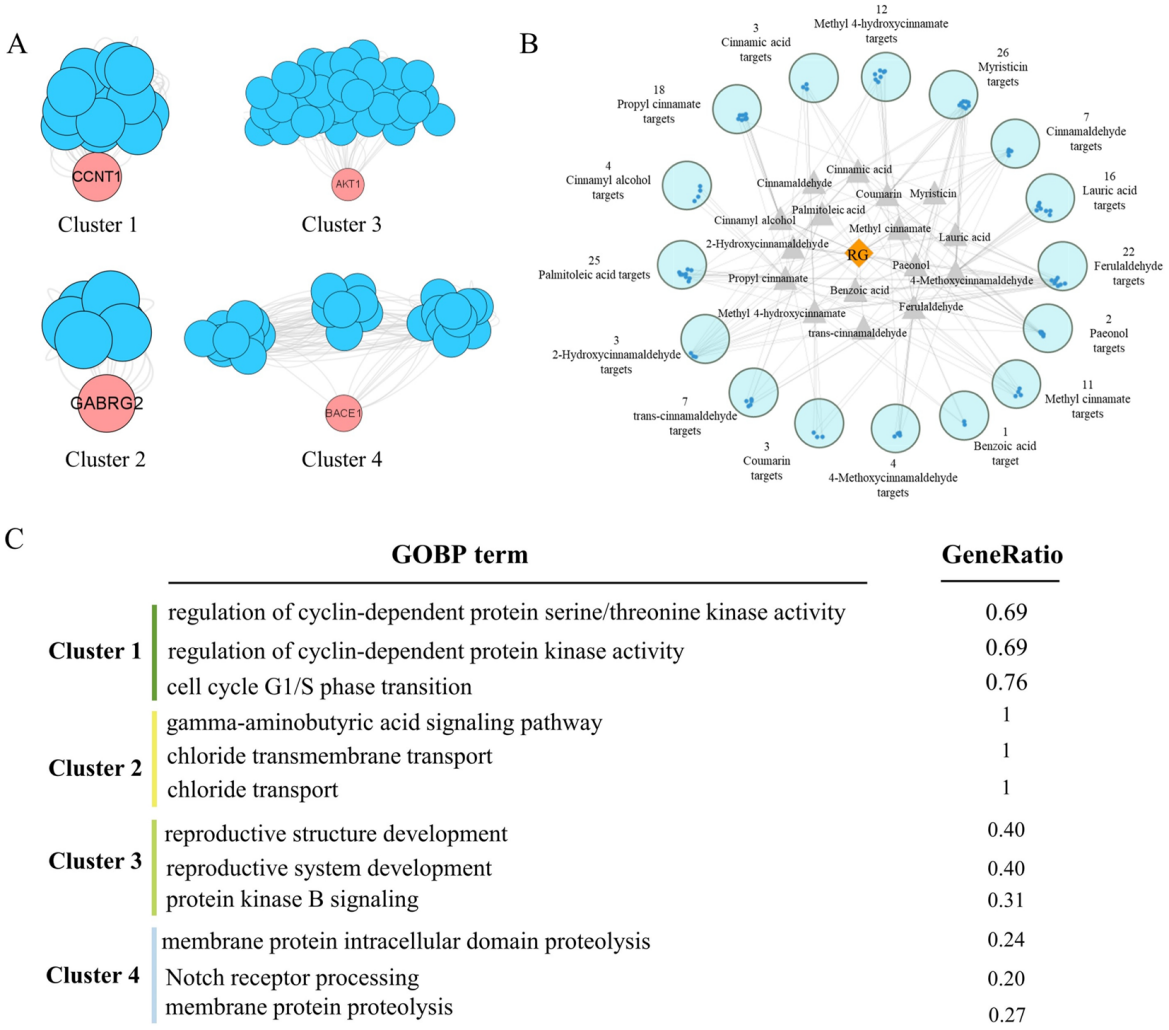
Clustering analysis of cinnamon brain-entry targets. (**A**) Four clusters of cinnamon brain-entry targets. (**B**) PPI network diagram of brain-entry targets with corresponding targets. (**C**) GO-BP analysis corresponding to each cluster.

**Figure 6:**
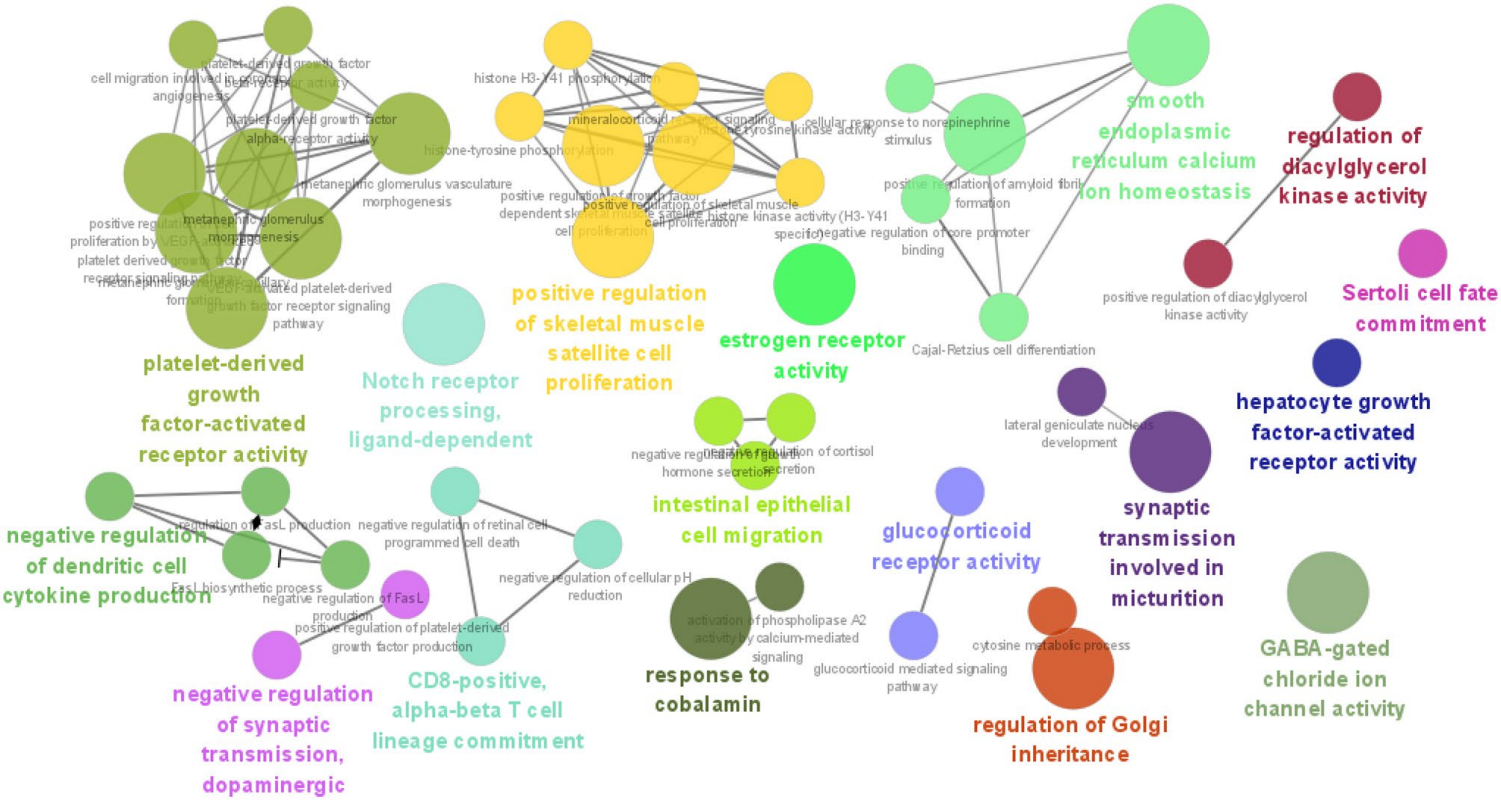
Pathways of brain active chemical compositions based on bioinformation analysis.

Enrichment analysis of 169 targets for compounds with BBB values greater than 0.3 was performed to determine PPI network function clusters using the MCODE plugin. The basic parameters aim to construct a corresponding meaningful list of clusters presented (Fig. 5A). The number of compounds and targets corresponding to the targets obtained by clustering was made to display (Fig. 5B). Cluster 1 (score: 12.333) consists of 13 nodes, seeded by CCNT1, also known as cell cycle protein T1, which encodes a highly conserved member of the C subfamily of cell cycle proteins, and the GO annotations associated with this gene include chromatin binding, transcriptional cis-regulatory region binding and processes, such as regulation of cyclin-dependent protein serine/threonine kinase activity, regulation of cyclin-dependent protein kinase activity, etc. Cluster 2 (score: 7.000) consists of 7 nodes, whose seed gene is GABRG2, also known as γ-aminobutyric acid type A receptor subunit Gamma2, and the GO annotations associated with this gene include chloride channel activity and GABA-A receptor activity. Cluster 3 (score: 6.647) consists of 35 nodes, the seed gene of which is AKT1, also known as AKT serine/threonine kinase 1. The GO annotations associated with this gene include the same protein binding and protein kinase activity, and the GO pathway involved in this cluster is mainly involved in reproductive structure development, etc. Cluster 4 (score: 5.571) consists of 29 nodes, the seed gene is BACE1, also known as β-secretase 1, which encodes a member of the A1 family of aspartate proteases. Notch signaling pathway and other processes such as membrane protein intracellular domain proteolysis, Notch receptor processing, amyloid precursor protein catabolic process, etc. process, etc. Finally, the GO-BP results of each cluster are shown (Fig. 5C). The cinnamon in brain targets were intersected with AD targets, and the 169 targets obtained were converted into gene IDs and imported into clueGO for displaying the results (Fig. 6).

### Association analysis and network construction

**Figure 7:**
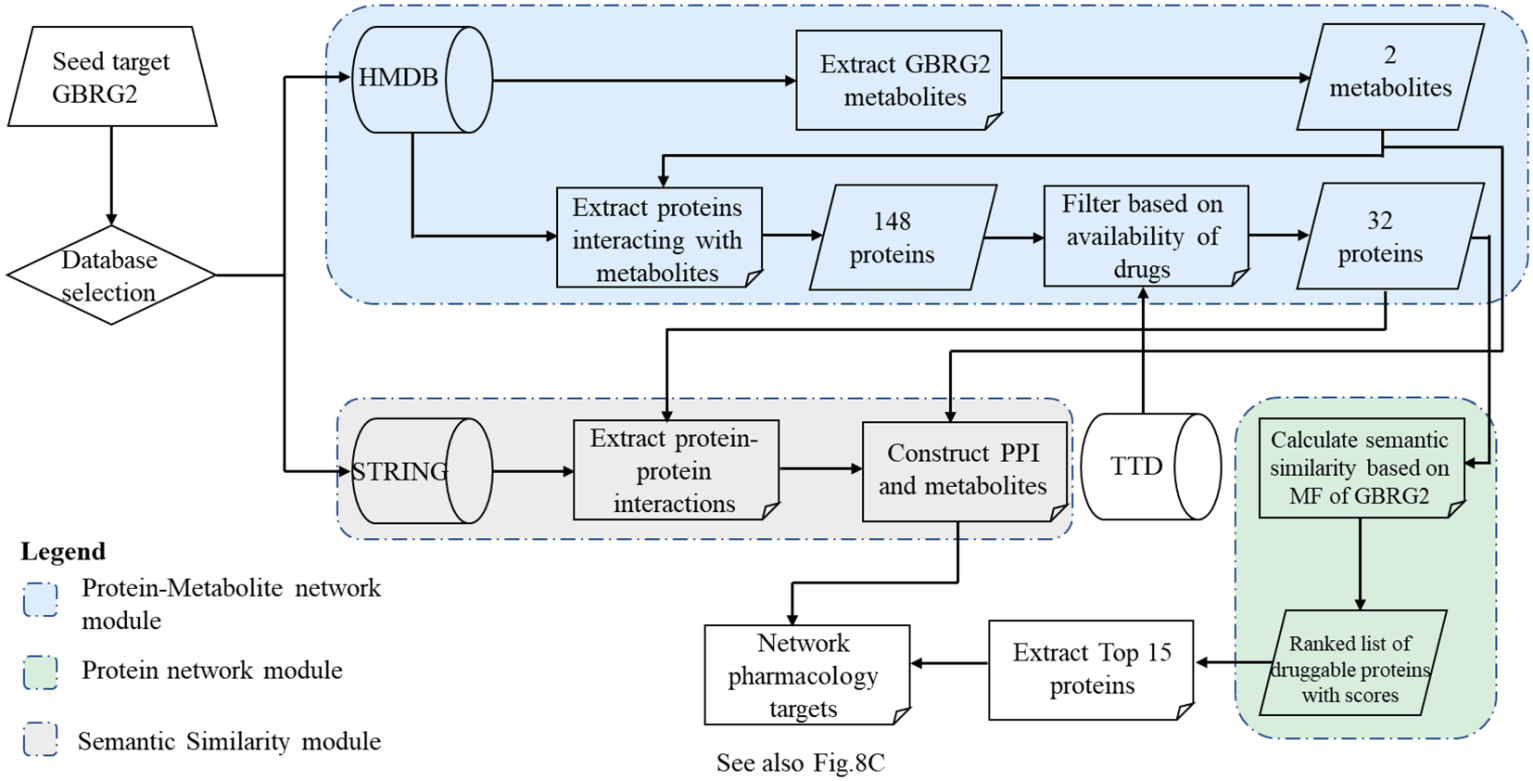
Computational procedure for prioritization of targets through network pharmacology. The pipeline for calculating target prioritization composes of 3 symbiotic blocks. The blue block searches metabolites from the Human Metabolome Database (HMDB) that interact with the GBRG2 protein, collates the metabolites, obtains the targets that interact with them, and screens them against drug availability in the Therapeutic Target Database (TTD). The grey module uses the STRING database to extract PPI from the proteins generated by the blue module and constructs a network from them. The green module uses molecular function (MF) annotations to calculate a GO-based semantic similarity score for the export of the blue module compared to GBRG2, ranking the proteins according to their similarity scores. The output of the green module was used to annotate the top 15 proteins in the network.

**Figure 8:**
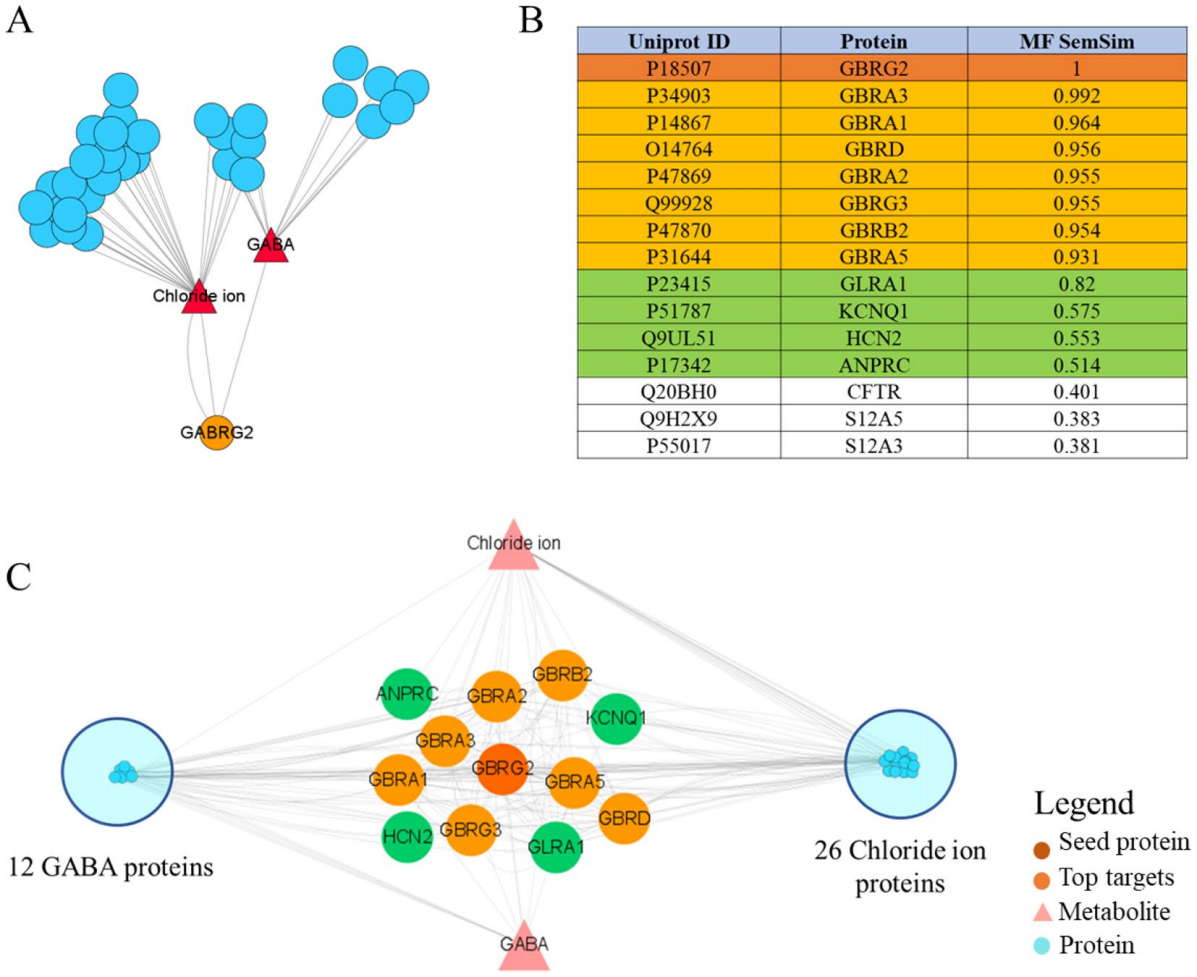
Integrated GBRG2-expanded multilayer biomolecular interaction network for candidate extraction and semantic similarity ranking of the proteins involved. (**A**) The complete network constructed using the major protein GBRG2 (orange nodes), linked to its direct metabolites (red nodes) which are linked to the proteins they interact with (blue nodes). We also show the metabolite-protein interactions (grey edges). (**B**) Semantic similarity ranking of proteins based on molecular function (MF SemSim) is highlighted, with the first 15 similar proteins. (**C**) The simplified network has only the main protein, with the top 12 similar proteins and metabolites shown separately, while the remaining proteins are grouped into modules and their interactions are merged.

To identify homologous targets of GBRG2, we used a case-by-case association analysis of multilayer molecular interaction networks. Signaling events mediated by intermediate metabolites play a greater role in the search for secondary targets than relying solely on PPI. Therefore, we combined PPI with protein-metabolite interactions to overcome this unknown prejudice or restriction. A bottom-up approach consisting of three interactive computational modules was employed, starting with one target affecting AD disease, GBRG2, as our primary target protein and seed node (Fig. 7). In module 1, this seed node is expanded to obtain a network of candidate targets and associated metabolites, yielding 2 meaningful metabolites, and then expanded to their interactors, yielding 148 proteins. As a filtering step to narrow the interaction search scale, we used drug-target interactions, yielding 32 potentially druggable targets. According to the preceding attained druggable target proteins, a PPI network has been constructed in the second module (Fig. 7). Next, the networks of module 1 and module 2 were integrated to attain a bilayer network that identifies the nearest interaction subjects of the primary targets by association. Thus, several levels of association with GBRG2 were found through straight protein interventions or indirect metabolite interference (Fig. 8A).

### Semantic similarity of GO terms confirms network analysis results

Semantic similarity is a metric defined over a set of documents or terms. It is used in this article to evaluate the functional annotation function of GO molecules for different targets^19^. In the flow chart in Fig. 7, Module 3 shows the GO term similarity scores corresponding to each target as a means of comparing the functional relevance of the two proteins. To score the term pairs, we utilized the method of Wang et al.^20^ because it can deduce similarity based on the GO hierarchy rather than just the direct terms in the alignment. The best average matching strategy, a method that not only considers only similar as well as dissimilar terms but is also less influenced by the figure of terms for comparison, can cling these scores into a functional relevance score for both proteins^19^. The inference that protein functionally similar to GBRG2 can be collated based on the GO similarity score is derived from the hypothesis that protein function can be used as a delegate for structural and biological similarity. In the last, the results of the semantic analysis were intersected with the list of cinnamon and AD disease intersection targets to narrow the list of candidate targets to only 5. GBRA3, GBRA1, GBRA2, GBRB2, GBRA5 were collated as the most functionally similar drug targets with similarity scores of 0.992, 0.964, 0.955, 0.954 based on GO annotated molecular functions, respectively, 0.931 (Fig. 8B and C). By using hybrid protein metabolic network analysis, a close association between GBRG2 and the GABA-A receptor family was predicted, and we next wanted to validate our findings using molecular docking, which would apply to network pharmacology.

### Molecular docking

**Figure 9:**
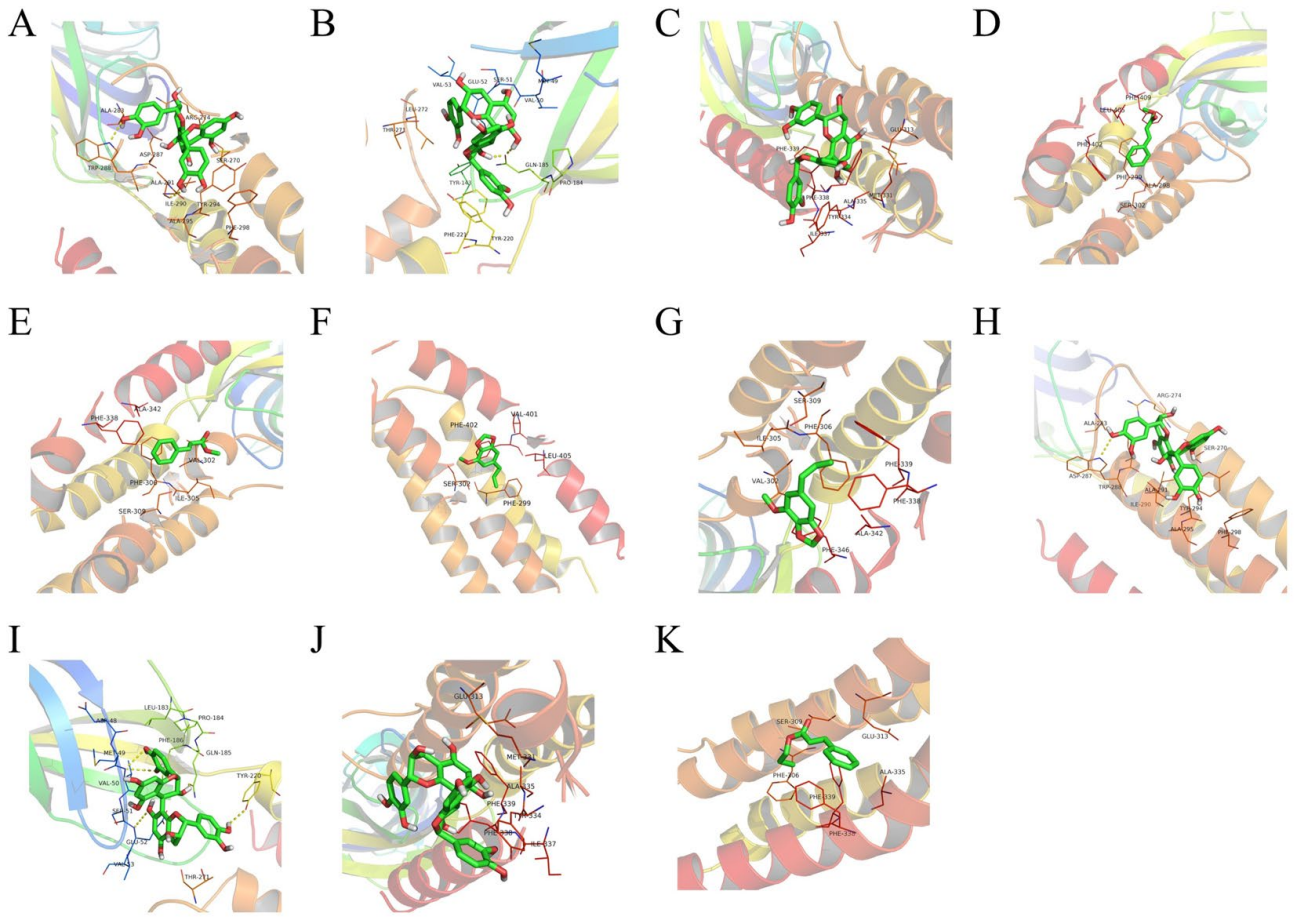
3D docking of the active ingredient (ligand) of cinnamon aqueous extract against AD-related GABAergic synaptic dysfunction targets (receptors) (**A**-**K**) The binding effect of ligand and receptor are shown.

Molecular docking aims to forecast the interactions between cinnamon active compounds and the homographic target proteins. For example ( +)-Procyanidin B2 with GABRA1, GABRB2, and GABRG2, Methyl cinnamate with GABRA5 and GABRG2, Myristicin with GABRA5 and GABRG2, Procyanidin B1 with GABRA1, GABRB2 and GABRG2. Propyl cinnamate has a potential high affinity with GABRG2. The two-dimensional binding patterns of bioactive components and proteins are listed in Fig. 9 and Table 2.

**Table 2:** Interaction between the compounds and the protein targets.

Number	Ligand	Receptor	Affinity (kcal/mol)	Interactions
A	( +)-Procyanidin B2	GABRA1	− 7.1	Hydrophobic interaction: Ala283, Asp287, Tyr294, He290, Phe298, Ala291; Hydrogen bond: Trp288, Ser270, Ala291, Ile290
B	( +)-Procyanidin B2	GABRB2	− 6.8	Hydrophobic interaction: Val53, Glu52, Thr271, Tyr220, Tyr143, Phe221, Pro184, Met49, Ser51, Val50, Leu272; Hydrogen bond: Gln185
C	( +)-Procyanidin B2	GABRG2	− 6.7	Hydrophobic interaction: Glu313, Phe339, Ala335, Tyr334, He337, Phe338, Met331
D	Methyl cinnamate	GABRA5	− 5.1	Hydrophobic interaction: Ser302, Ala298, Phe402, Phe299, Leu405
E	Methyl cinnamate	GABRG2	− 5.3	Hydrophobic interaction: Phe338, Phe306, Ser309, He305, Val302, Ala342
F	Myristicin	GABRA5	− 5.2	Hydrophobic interaction: Phe299, Ser302, Phe402, Leu405, Val401
G	Myristicin	GABRG2	− 5.1	Hydrophobic interaction: Phe339, Phe306, Val302, He305, Phe346, Ala342, Ser309, Phe338
H	Procyanidin B1	GABRA1	− 7.0	Hydrophobic interaction: Arg274, Ala283, Asp287, Tyr294, Ala295, Phe298, He290; Hydrogen bond:Trp288, Ser270, Ala291
I	Procyanidin B1	GABRB2	− 6.9	Hydrophobic interaction: Val53, Ser51, Glu52, Phe186, Met49, Leu183, Pro184; Hydrogen bond: Tyr220, Thr271, Val50, Asp48, Gln185
J	Procyanidin B1	GABRG2	− 6.7	Hydrophobic interaction: Met331, Phe338, He337, Tyr334, Ala335, Phe339, Glu313
K	Propyl cinnamate	GABRG2	− 5.0	Hydrophobic interaction: Glu313, Ala335, Phe306, Phe338, Phe339; Hydrogen bond: Ser309

## Discussion

AD is an age-dependent neurodegenerative disease that causes dementia primarily in the elderly, accompanied by symptoms of cognitive impairment, memory imbalance and behavioral abnormalities. More than 50 million people worldwide are currently diagnosed with AD, and this number is expected to increase significantly to 152 million in 2050 with the advent of an aging society. Statistics estimate that one person worldwide is diagnosed with AD every three seconds^21–23^. However, the greatest challenge at present is to discover potential target proteins involved in anxiety-like behaviors resulting from AD-related synaptic impairment to construct a much more accurate therapeutic approach that not only controls the further progression of the pathology but also promotes the remission or resolution of clinical symptoms. Given that patients suffering from AD are mostly associated with mood disorders such as anxiety and irritability, which greatly affect the cognitive decline of AD patients. Cinnamon is multi-targeted and multi-effective and is widely used in treating AD because of its antioxidant, anti-inflammatory, antibacterial, anti-anxiety and antidepressant properties^24^. In the current study, we hope to confirm these targets by systemic pharmacological assessment of those proteins regulated by cinnamon that could be medicinable targets of this traditional Chinese medicine to counteract the anxiety observed in AD patients. Overall, our results suggest that GABRA1, GABRB2, GABRA5 and GABRG2 are proteins that are especially influenced by this cinnamon and that they could play a key action in this pathological process.

In this study, we found that the GABA signaling pathway contains key targets regulated by cinnamon aqueous extract and is altered due to AD. It is well known that AD patients are accompanied by varying degrees of anxiety, which in turn can impair cognitive function in the brain. The core brain area of the anxiety neural circuit is the amygdala, which is responsible for receiving signals from the cortex and thalamus and then regulating the production of fear and anxiety-related behaviors. Inhibitory control dominates information processing in the amygdala^25^. Rapid inhibitory neurotransmission in the brain is primarily mediated by the neurotransmitter GABA and its synaptic target GABA receptor type A (GABAA receptor)^26^. GABAA receptors are ligand-gated ion channels composed of α1-6, β1-3, γ1-3, δ, ε, theta, and π subunits to form a heterotetrameric transmembrane protein complex^27^. However, the literature increasingly supports the idea that persistently low concentrations of GABA in the cytoplasm can activate those GABAA receptor subtypes distant from the synapse to generate “tonic” conductance^28^. GABAergic transmission is deficient in anxiety^29^. Neurons expressing GABAA α1 receptors can mediate sedation, while those expressing GABAA α2 receptors mediate anxiolytic. In addition, extra-synaptic GABAA α5 receptors can also regulate the activity of hippocampal pyramidal cells, thereby affecting associative temporal and spatial memory^30^. The literature reports that environmental changes in rodents early after birth lead to long-term changes in responses to stress that may underlie anxiety. During this period, the subunit composition of GABAA receptors changed significantly, such as the regio-dependent replacement of α2 with the α1 subunit^31^. Increased GABA levels in the amygdala of high anxiety-related behavior (HAB) mice. Relative to the control group, GABAA receptors β2 and γ2 subunits were notably improved in the basolateral amygdala, whereas transcription of the α5 and γ1 subunits was decreased in the central and medial amygdala. In HAB mice, the basolateral amygdala has observed a noticeable increase in levels of β2 and γ2 subunit immunoreactivity through Western blot experiments^32^. The specific mechanism, anxiety-related behaviors modulated by amygdala GABAergic neurons and synapses, haven’t been elucidated^25^. But to be sure, much evidence reveals that disruption of the default neuronal network underlies memory impairment and results in marked alterations in GABAergic circuits, either primarily or as a compensatory response to excitotoxicity, and by disrupting overall network function^2^.

It is worth noting that the four most significant targets, GABRA1 (GABAA receptor α1 subunit), GABRB2 (GABAA receptor β2 subunit), GABRA5 (GABAA receptor α5 subunit) and GABRG2 (GABAA receptor γ2 subunit) obtained from the final screening of cinnamon aqueous extract, were significantly enriched in the GABA signaling pathway, which may illustrate a dominant part of cinnamon aqueous extract in inhibiting AD-related GABAgeric synaptic dysfunction. Molecular docking is a theoretical simulation method that mimics ligand-receptor interactions and predicts their binding patterns and affinities. To identify the interference between cinnamon aqueous extract efficient components and targets, molecular docking experiments were performed. For example, ( +)-Procyanidin B2 and Procyanidin B1 have high affinity with GABRA1, Methyl cinnamate and Myristicin have a potential high binding affinity with GABRA5 and GABRG2, etc. It has been reported in the literature that black hawthorn fruit, asparagus, and cocoa extracts, whose main component is procyanidins, reduce anxiety/depression symptoms in rats^33–35^. Xiao et al. demonstrated that the administration of Procyanidin B2 from lotus in insomniac rat models was accompanied by an increase in the concentration of γ-aminobutyric acid in the brain and prolonged sleep time in rats^36^. Cinnamaldehyde is the main active component of cinnamon aqueous extract, which may be further converted to cinnamyl alcohol, methyl cinnamate and cinnamic acid in vivo^8^. Methyl cinnamate was reported to be one of the active components of the standardized ethanolic extract of EPMG, and the findings suggest that the anxiolytic effect displayed by the ethanolic extract of EPMG may be mediated by the GABAergic system^9^. In a potential interaction with the GABA receptor in male Sprague–Dawley rats regarding the anxiolytic effect of the main compound myristicin, the results confirmed that myristicin does not reduce anxiety by modulating GABAA receptors, but may promote angiogenesis. When myristicin was combined with midazolam, anxiety decreased compared to the midazolam-only group, exhibiting an antagonist effect similar to that of the combination of flumazenil and myristicin^37^.

Since anxiety-like emotions in AD can lastingly affect the worsening of the disease course, research aimed at exploring new treatments has not only received high attention from a clinical perspective, but has also opened up new alterations as hope for reducing the loss of life of AD patients affected by anxiety. The “one disease-one target-one drug” method is widely accepted in drug discovery, as a result, streamlines the compound screening, simplifies the process of registration as well as circumvents side effects^38^. However, the above-mentioned method oversimplifies disease mechanisms, which are actually complicated sub-networks in the interaction set^39^. Furthermore, the definition of disease is mostly based on symptoms rather than mechanisms, and therefore the same is true for treatments. Therefore, It is the reason that drug discovery is currently assumed to be progressively inefficient^40^. In contrast, causal mechanisms are the main principles of systems medicine and network pharmacology to identify diseases^41^. Furthermore, network pharmacology intends to enhance this trait, not only by targeting individual components of such networks but also by exploiting drugs within these networks to achieve synergistic effects or dose reduction^42^. In short, through network pharmacology and enrichment analysis, we screened five components, methyl cinnamate, propyl cinnamate, ( +)-procyanidin B2, procyanidin B1, and myristicin as the brain synapse-targeting active substances of cinnamon, and identified GABRA1, GABRB2, GABRA5 and GABRG2 as core therapeutic targets of cinnamon against Alzheimer’s disease-related GABAergic synaptic dysfunction. Exploring the mechanism of cinnamon’ activities through multi-components and multiple targets strategies promise to reduce the threat of single-target and symptom-based drug discovery failures.

On the one hand, the parameters of cinnamon chemical components entering the BBB collected in the database may also be inaccurate. On the other hand, cinnamon undergoes complex metabolism during the in vivo process before it acts, and the compounds that end up in the brain are not necessarily prototypes, while our study used a network pharmacology approach to pre-calculate the possible anti-AD-related anxiety effects of cinnamon using the condition of whether the prototype compounds cross the BBB. In addition, as the database is updated and the site algorithm is optimized, it can limit our pre-curated results. In future studies, it may be possible to search for secondary metabolites contained in TCM or identify secondary metabolites of TCM through in vivo metabolism of drugs by using big data analysis theory and mathematical and statistical methods, and identify active small molecules by comparing published literature. Furthermore, we should identify the components of cinnamon that are metabolized in vivo, especially into the brain, and validate the results based on swiss target prediction and molecular docking predictions using molecular biology.

## Materials and methods

### Preparation of cinnamon aqueous extracts

The Cinnamon herb was provided by Shanghai Kangqiao Chinese Herb Slices Co., Ltd. (Cat No.210203, Shanghai, China) and identified as *Cinnamomum cassia Presl* by Ph.D Ting Zhang. This study complies with relevant institutional, national, and international guidelines and legislation. The cinnamon aqueous extract was improved according to the method of Elshopakey et al.^43^. The cinnamon samples were crushed and sieved with 50 mesh. 10 g of cinnamon sample was soaked in distilled water (100 mL). The mixture was boiled at 100 °C for 2 h, then filtered and dried overnight by heating in the 80 °C water bath. The resulting dry extract was weighed and reserved for further analysis and processing. According to the literature, 50% methanol has good solubility for most of the active ingredients in cinnamon, such as caffeic, ferulic, p-coumaric, protocatechuic and vanillic acids. Therefore, 50% methanol was chosen to solute the extract^44^. Then 200 mg of the extract was dissolved in 2 mL of 50% methanol solution, sonicated, and fixed in a volumetric flask.

### LC–MS analysis

Before the Ultimate 3000/Easy nLC 100-LTQ-Orbitrap Elite LC analysis, 1 mL of supernatant was filtered through a 0.22 μm microporous filter. LC was performed by the Ultimate 3000 system, and mass spectrometry was performed by the Easy nLC 100-LTQ-Orbitrap Elite system. Liquid phase conditions: Waters Acquity UPLC HSS T3 column (2.1 × 100 mm, 1.8 μm) was rinsed with a mobile phase of 0.1% formic acid water—acetonitrile (A–B) and a gradient elution (0–2 min, 2–5% B; 2–13 min, 5–50% B; 13–25 min, 50–95% B; 25–30 min, 95% B; 30–31 min, 95–2% B; 31–35 min, 2% B); flow rate: 0.3 mL/min, column temperature: 40 °C, injection volume: 4 μL.

Mass spectrometry conditions: ESI ion source, positive and negative ion mode detection, spray voltage of 3.8 kV, heater temperature of 300 ℃, sheath gas volume flow rate of 35 arb, auxiliary gas volume flow rate of 15 arb, tail gas volume flow rate of 0.1 arb, the capillary temperature of 350 ℃, sheath gas and auxiliary gas are nitrogen. The scanning mode was full scan mode, and the scanning scale was from 50 to 1000 m/z, with a collision energy of 50 V.

### Identification of the chemical composition of cinnamon aqueous extract from the database

Thermo Xcalibur Qual Browser software (Thermo, USA) was used to detect the HPLC/MS results. With the Easy nLC 100-LTQ-Orbitrap Elite system, the mass accuracy of all obtained ions was no more than 5 ppm, and their exact isotopic patterns can be confirmed. Therefore, molecular formulae were easily identified by accurate mass and isotope enrichment ratios using the “formula finding function” of the Thermo Xcalibur Qual Browser software. The fragmentation information of compounds with parallel structures could be studied, then these rules were used for the structural identification of derivatives with the general structure. To identify the compound structures, the accurate masses of MS/MS fragments were input to and compared with compounds in the database of mzVault, mzCloud, ChemSpider, and literature. The compounds from HPLC /MS were screened in the TCM database based on mass-to-charge ratio, i.e. TCMSP (http:// tcmspw.com/tcmsp.php)^45^. The cross-components of HPLC/MS and TCM database are considered as the main potential active compounds for further investigation of network pharmacology.

### Target predictions related to the components identified by cinnamon aqueous extracts

To explore the valuable targets of the filtered compounds, a search was performed in the PubChem database (https://pubchem.ncbi.nlm.nih. gov/)^46^. SMILES and SDF format files of the above components were retrieved to build a collection of molecules for screening. The SMILES files of the confirmed components were uploaded to the Swiss Target Prediction database (http://www.swisstargetprediction.ch/)^47^. Information on the targets equivalent to the components is acquired. The obtained protein targets were deduplicated and submitted to the UniProt database (https://www.uniprot.org) for conversion into gene symbols.

### Screening cinnamon aqueous extracts targets based on blood–brain barrier (BBB) permeability

SMILES were imported the into Swiss ADME website^48^ to obtain the GI and BBB values of the compounds. Gastrointestinal absorption (GI absorption) was set to “High” as the screening criteria for drug absorption to filter bioactive components with better oral bioavailability. The BBB value was set to “Yes” as a condition for drug entry into the BBB for the screening of compounds with good central activity. The TCMSP database was used to obtain the OB and BBB values of the compounds, where the compounds with BBB values greater than 0.3 had strong blood–brain permeability. The cinnamon targets collected after screening were entered into the Search Tool for the Retrieval of Interacting Genes (STRING) database^49^ for PPI network analysis, and then the results were imported into Cytoscape 3.8.1 software for MCODE clustering analysis^50^. Proteins were annotated using GO terms and KEGG (https: //www.kegg.jp/) terms to study identified cinnamon targets at the functional level^51^. Parameters were set with *p* < 0.05, species were confirmed as “Homo sapiens”, and target genes were defined with their official gene symbols. To explore drug actions in vivo, we retained the most meaningful GO pathways in the enriched genes, and then visualized the results using the Microbiotope website.

### Enrichment analysis of common targets of cinnamon aqueous extracts and AD

Using Genecards, Drugbank, OMIM, PharmGkb, and TTD, a target library with “Alzheimer's disease” as a keyword was generated. First, all the targets of cinnamon were intersected with the AD targets. We used the online tool Venny 2.1.0 to obtain the intersection of cinnamon and AD targets, and imported the intersection the targets into string website to get the interaction between the targets. GO and KEGG pathway analyses were utilized to elucidate further the biological interpretation of key genes in the core network. Further understanding of higher-order functions of biological systems relies primarily on gene classification and enrichment analysis, based on the software of 32 new ontology-based packages clusterProfiler, version R3.6.0. Biological Process (BP), Molecular Function (MF), and Cellular Component (CC) are the three parts of GO, and the top 15 GO analysis terms were selected and further visualized using the website of Microbiology Letter. In addition, bubble plots were also used to present the top 15 KEGG enrichment analyses, where smaller *P* values were marked in red, larger *P* values were indicated in blue, and the size of the dots represented the gene proportions. Then, the cinnamon active compounds with BBB values greater than 0.3 were then intersected with AD targets, and the pathway enrichment analysis was performed on Cytoscape 3.8.1 software using Cluego, where the screening condition for Cluego was *p* < 0.01, GO-BP process.

### Mechanistic analysis of the anti-AD target of cinnamon aqueous extracts

GABRG2 is considered a core target of cinnamon clustering targets and a relevant target and primary target for GABA synaptic disorders in AD diseases. According to the UNIPROT database, its protein name is GBRG2. Integration of the two the databases, STRING database for PPI and the Human Metabolome Database (HMDB), was performed to study protein-metabolite interactions covering two data sources based on the possibility that proteins interact directly (physical PPI) or indirectly (downstream reactions through metabolites). Since related proteins have been found to regulate immediately (physical PPI) or indirectly (activation of downstream interactions by metabolites), we chose to consolidate both databases for protein-metabolite interactions. STRING database can consolidate given and predictive relationships among proteins, such as physical interplay and functional relevance. To realize this destination, STRING gathers and grades basis on multiple approaches: databases of interaction experiments and annotated complexes/pathways, automated text mining of the scientific literature, computational interaction predictions from co-expression, and systematic transfer of evidence from conserved genomic context and interactions from one organism to another^49^. Just as follows, we constructed a two-tier network by integrating STRING and HMDB. First of all, we selected metabolites that have interaction with GBRG2, a pivotal target. We then extracted all proteins that interact with these metabolites, generating a two-tier protein/metabolite network centered on GBRG2. Proteins that were annotated as known drugs were screened from the network as clinically relevant targets were more valuable. The Therapeutic Target Database (TTD) is used to obtain a list of druggable proteins to this end. Next, direct molecular interactions between them are used to construct a network of potential targets. Nevertheless, the result only offers a manner to assess the connectivity of proteins to our primary proteins, rather than a quantitative method to evaluate the functional similarity of the potential synergistic genes. Therefore, we utilized semantic analysis to collate the linked proteins orderly. We used the GOSim R package, a strategy for scoring the similarity of each protein's GO terms to the molecular function of GBRG2. Wang et.al designed an algorithm for evaluating the semantic similarity of GO terms with an evaluation value between 0 and 1. This algorithm is calculated based on the position of the GO terms corresponding to different genes in a directed acyclic graph (DAG)^40^. It confirms the semantic score of each term according to the total of the weighted contributions of all ancestors, where the closer ancestor term has a bigger score. After searching the two proteins with the affiliated all GO terms, semantic similarity of GO terms can be used to evaluate the resemblance between two targets. In the last, we visualized the extended network and the reduced network with an open-source freeware Cytoscape 3.8.1 software (https://cytoscape.org/)^52^ and annotated the top targets^53^.

### Molecular docking studies

The obtained ligand–protein interactions were analyzed with AutoDock Vina. The results of docking are displayed by PyMOL software (Version 1.7.0, https://pymol.org/). The crystal structures of GABRA1 (PDB: 6CDU), GABRA5 (PDB: 5OBF), GABRB2 (PDB: 6X3W) and GABRG2 (PDB: 6X3W) were obtained from the RCSB protein database, with all polar hydrogen atoms attached and solvation storage parameters allocated. During the docking process, the substructures of the ligands were summarized and water molecules were removed. The overall grade is presented as affinity and represents the binding affinity; the larger the overall docking grade, the better the interaction between the protein and the bioactive compound.

## Data Availability

The datasets used and/or analysed during the current study available from the corresponding author on reasonable request.

## Abbreviations

Aβ: β-Amyloid peptide
AD: Alzheimer’s disease
BBB: Blood brain barrier
BP: Biological process
CC: Cellular components
CNS: Central nervous system
DAG: Directed acyclic graph
EPMG: Ethanolic extract of mikania glomerate
GABA: γ-Aminobutyric acid
GO: Gene ontology
HAB: High anxiety-related behavior
HMDB: Human metabolome database
KEGG: Kyoto encyclopedia of genes and genomes
MCI: Mild cognitive impairment
MF: Molecular function
NFTs: Neurofibrillary tangles
OMIM: Online mendelian inheritance in man
PharmGkb: Pharmacogenetics and pharmacogenomics knowledge base
STRING: Search tool for the retrieval of interacting genes
TCMSP: Traditional Chinese medicine systems pharmacology database and analysis platform
TTD: Therapeutic target database
UPLC-Q-TOF–MS/MS: Ultra-performance liquid chromatography-quadrupole-time-of-flight tandem mass spectrometry
